# Decoding the Genomic Profile of the *Halomicroarcula* Genus: Comparative Analysis and Characterization of Two Novel Species

**DOI:** 10.3390/microorganisms12020334

**Published:** 2024-02-05

**Authors:** Dáša Straková, Cristina Sánchez-Porro, Rafael R. de la Haba, Antonio Ventosa

**Affiliations:** Department of Microbiology and Parasitology, Faculty of Pharmacy, University of Sevilla, 41012 Sevilla, Spain; daska.strakova@gmail.com (D.S.); sanpor@us.es (C.S.-P.); rrh@us.es (R.R.d.l.H.)

**Keywords:** haloarchaea, hypersaline soil, taxogenomic analysis, comparative genomic analysis, pan-genome

## Abstract

The genus *Halomicroarcula*, classified within the family *Haloarculaceae*, presently comprises eight haloarchaeal species isolated from diverse saline habitats, such as solar salterns, hypersaline soils, marine salt, and marine algae. Here, a detailed taxogenomic study and comparative genomic analysis of the genus *Halomicroarcula* was carried out. In addition, two strains, designated S1CR25-12^T^ and S3CR25-11^T^, that were isolated from hypersaline soils located in the Odiel Saltmarshes in Huelva (Spain) were included in this study. The 16S rRNA and *rpoB’* gene sequence analyses affiliated the two strains to the genus *Halomicroarcula*. Typically, the species of the genus *Halomicroarcula* possess multiple heterogeneous copies of the 16S rRNA gene, which can lead to misclassification of the taxa and overestimation of the prokaryotic diversity. In contrast, the application of overall genome relatedness indexes (OGRIs) augments the capacity for the precise taxonomic classification and categorization of prokaryotic organisms. The relatedness indexes of the two new isolates, particularly digital DNA–DNA hybridization (dDDH), orthologous average nucleotide identity (OrthoANI), and average amino acid identity (AAI), confirmed that strains S1CR25-12^T^ (= CECT 30620^T^ = CCM 9252^T^) and S3CR25-11^T^ (= CECT 30621^T^ = CCM 9254^T^) constitute two novel species of the genus *Halomicroarcula*. The names *Halomicroarcula saliterrae* sp. nov. and *Halomicroarcula onubensis* sp. nov. are proposed for S1CR25-12^T^ and S3CR25-11^T^, respectively. Metagenomic fragment recruitment analysis, conducted using seven shotgun metagenomic datasets, revealed that the species belonging to the genus *Halomicroarcula* were predominantly recruited from hypersaline soils found in the Odiel Saltmarshes and the ponds of salterns with high salt concentrations. This reinforces the understanding of the extreme halophilic characteristics associated with the genus *Halomicroarcula*. Finally, comparing pan-genomes across the twenty *Halomicroarcula* and *Haloarcula* species allowed for the identification of commonalities and differences between the species of these two related genera.

## 1. Introduction

Hypersaline soils are terrestrial ecosystems characterized by their high salt content, showing an electrical conductivity higher than 4 mS/cm [1]. However, their microbiota can be influenced by various factors beyond salt concentration, including temperature, pH, and the presence of high levels of toxic substances [2,3]. The Odiel Saltmarshes, a protected natural area located in southwestern Spain, harbor extensive areas of hypersaline soils, influenced by the confluence of the Odiel and Tinto rivers and the presence of tidal waters. This area contains elevated concentrations of metals (arsenic, cadmium, copper, lead, zinc) due to natural geological processes, such as accumulation through evaporation, and due to the historical metallurgical operations in this region [4,5].

The genus *Halomicroarcula* belongs to the family *Haloarculaceae*, order *Halobacteriales* and class *Halobacteria*, within the phylum *Methanobacteriota* (formerly “*Euryarchaeota*”), and it was first described in 2013 by Echigo et al. [6]. It currently contains eight halophilic species: *Halomicroarcula pellucida* (type species), *Halomicroarcula amylolytica*, *Halomicroarcula laminariae*, *Halomicroarcula limicola*, *Halomicroarcula marina*, *Halomicroarcula nitratireducens*, *Halomicroarcula rubra*, and *Halomicroarcula salinisoli* [7]. Besides, the species *Halomicroarcula salina* has been recently transferred to the genus *Haloarcula*, as *Haloarcula salina* [8]. *Halomicroarcula* species have been isolated from diverse habitats, such as solar salterns, saline soils, solar salt, marine seaweed, and salt mines [6,8,9,10,11,12]. They are extremely halophilic, showing optimal growth at 20–25% (*w*/*v*) NaCl. The cells of the species are motile, Gram-stain-negative, and pleomorphic. They are aerobic or facultatively anaerobic microorganisms [6]. *Halomicroarcula* species exhibit pink, red, or orange pigmentation or can be transparent [12]. The major polar lipids are phosphatidylglycerol (PG), phosphatidylglycerol phosphate methyl ester (PGP-Me), and phosphatidylglycerol sulfate (PGS). Some species additionally possess a glycolipid chromatographically identical to sulfated diglycosil diether (S-DGD-1) and mannosyl glucosyl diether (DGD-1) [6,9].

In this study, a comparative genomic analysis, insights into the ecological distribution across various hypersaline habitats, and a detailed taxogenomic study of the species of the genus *Halomicroarcula* as well as two new strains isolated from hypersaline soils of the Odiel Saltmarshes Natural Area located in Huelva (Southwest Spain) are presented. The new strains, designated as S1CR25-12^T^ and S3CR25-11^T^, are described as two new haloarchaeal species, based on the phylogenetic, phenotypic, and chemotaxonomic characterization and the whole genome analysis.

## 2. Materials and Methods

### 2.1. Soil Sample Analysis and Isolation/Cultivation Conditions of Haloarchaeal Strains

Strains S1CR25-12^T^ and S3CR25-11^T^ were isolated from a hypersaline soil located in the Odiel Saltmarshes, southwestern Spain, in July 2020 (Supplementary Figure S1). The physicochemical characteristics of the soil samples were examined, including pH and electrical conductivity, which were measured using a pH meter (CRISON BASIC 20) and a conductometer (CRISON 35+), respectively, after a 1:5 dilution. Additionally, soil samples, designated as 1C (37°12′26.6″ N 6°57′52.5″ W) and 3C (37°13′18.0″ N 6°57′44.8″ W), corresponding to the isolation places of the studied strains, were analyzed by Innoagral Laboratories, in Brenes (Sevilla, Spain) to assess their salinity-related ion contents and heavy metal concentrations determined by atomic absorption spectroscopy. Serial dilutions and plating techniques were carried out under sterile conditions, followed by incubation of the Petri dishes at 37 °C for up to 3 months to isolate strains. After successive subculturing, they were obtained in a pure culture. To isolate and cultivate the strains, Reasoner’s 2A (R2A) medium (Difco, Beirut, Lebanon) was used, with the following composition per liter: yeast extract (0.5 g), proteose peptone no. 3 (0.5 g), casamino acids (0.5 g), dextrose (0.5 g), soluble starch (0.5 g), sodium pyruvate (0.3 g), dipotassium phosphate (0.3 g), and magnesium sulfate (0.05 g) containing 25% (*w*/*v*) total salts (designated as 25% R2A medium). This was prepared by diluting a 30% (*w*/*v*) stock salt solution [13], and it had the following composition per liter: NaCl (195 g), MgCl_2_·6H_2_O (32.5 g), MgSO_4_·7H_2_O (50.8 g), CaCl_2_ (0.83 g), KCl (5.0 g), NaHCO_3_ (0.17 g), and NaBr (0.58 g). The pH of the medium was adjusted to 7.5 with 1 M KOH, and when necessary, solid media were prepared by adding purified agar to a final concentration of 2% (*w*/*v*). Cultures were preserved at −80 °C in 25% R2A liquid medium containing 40% (*v*/*v*) glycerol for long-term storage.

### 2.2. DNA Extraction, Amplification, and Sequencing

The Marmur methodology [14], modified for small volumes, was employed to extract the genomic DNA of strains S1CR25-12^T^ and S3CR25-11^T^. The Bio-Rad T100 Thermal Cycler was used for PCR reactions to amplify the 16S rRNA and *rpoB’* genes. For amplification of these genes, the universal archaeal primers ArchF and ArchR (5′-TTC CGG TTG ATC CTG CCG GA-3′, 5′-GGT TAC CTT GTT ACG ACT T-3′) [15,16], as well as the rpoBF and rpoBR (5′-TGT AAA ACG ACG GCC AGT TCG AAG AGC CGG ACG ACA TGG-3′, 5′-CAG GAA ACA GCT ATG ACC GGT CAG CAC CTG BAC CGG NCC-3′) primers were selected [17,18]. The integrity of the genomic DNA and PCR amplicons was verified by 1% (*w*/*v*) agarose gel electrophoresis. To purify the genomic DNA and PCR products, the MEGAquick-spin^TM^ Plus Fragment DNA Purification Kit (iNtRON Biotechnology, Seongnam, Republic of Korea) was used following the manufacturer’s instructions. The PCR reaction for amplifying the 16S rRNA gene involved denaturation of the prokaryotic DNAs at 95 °C for 5 min, followed by 25 cycles of denaturation at 94 °C for 1 min, primer hybridization at 50 °C for 1 min, and extension at 72 °C for 2 min. The reaction concluded with a final elongation step at 72 °C for 10 min. The DNA concentration was measured using the Qubit 4 Fluorometer (Thermo Fisher Scientific, Waltham, MA, USA), and the quality of the extracted DNA was assessed spectrophotometrically using the NanoDrop One spectrophotometer (Thermo Fisher Scientific). The PCR amplicons were sequenced using the Sanger chain-termination method by Stab Vida (Caparica, Portugal). Furthermore, Novogene Europe (Cambridge, UK) performed whole genome sequencing of strains S1CR25-12^T^ and S3CR25-11^T^ using the Illumina (San Diego, CA, USA) NovaSeq 6000 platform.

### 2.3. Phylogenetic and Phylogenomic Analyses

The ChromasPro software (Technelysium Pty Ltd., Brisbane, Australia), version 1.5, was used to assemble the 16S rRNA and *rpoB’* gene sequences of the isolated strains. These sequences were then compared with those available in the EzBioCloud database [19] and the NCBI GenBank database using the BLASTN search tool [20] to establish their taxonomic affiliation with phylogenetic neighbors. The alignment of the *rpoB’* gene sequences was performed using the BioEdit program v.3.3.19.0 [21]. The ARB software v.7.0 [22] was employed for phylogenetic tree construction based on the sequence data of the 16S rRNA and *rpoB’* genes. Maximum-likelihood [23], neighbor-joining [24], and maximum-parsimony algorithms [25] were used for phylogenetic tree reconstructions. The “gitana” script [26] was used for formatting and visualization of the phylogenetic trees. To estimate branch support in the phylogenetic treeing, a bootstrap analysis with 1000 replications was performed [27]. The 16S rRNA and *rpoB’* gene sequences of strains S1CR25-12^T^ and S3CR25-11^T^ were deposited in GenBank/EMBL/DDBJ under the accession numbers ON653025 and ON653386 (*rrnA*), ON653026 and ON653385 (*rrnB*), and ON668041 and ON668045 (*rpoB’*), respectively.

To conduct the taxogenomic analysis, genomic sequences of closely related species from the GenBank database were obtained, following the established guidelines for taxonomic use of genome data [28]. The genomic reads of strains S1CR25-12^T^ and S3CR25-11^T^ were subjected to k-mer assembly employing Spades v.3.13.0 [29], followed by verification of assembly integrity using CheckM v.1.0.5 [30] to ensure the absence of contamination. Subsequently, the standard genome annotation was performed using Prokka v.1.12 [31]. The whole-genome sequences of strains S1CR25-12^T^ and S3CR25-11^T^ were deposited in the GenBank/EMBL/DDBJ databases, and they were assigned the accession numbers JAMQON000000000 and JAMQOS000000000, respectively. The amplified 16S rRNA and *rpoB’* gene sequences obtained via PCR correlated with the sequences derived from genomic sequencing. The Enveomics toolbox [32] was used to identify clusters of orthologous proteins shared by all strains subjected to analysis. The approximately maximum-likelihood phylogenomic tree, based on 157 core-orthologous protein sequences, was reconstructed using FastTreeMP v.2.1.8, as described by Price et al. [33].

### 2.4. Comparative Genomic Analysis

The overall genome relatedness indexes (OGRIs) were computed to assess the genomic relatedness between strains S1CR25-12^T^ and S3CR25-11^T^, and the type strains of other species within the family *Haloarculaceae*. The orthologous average nucleotide identity (OrthoANI) values were determined using the OrthoANIu tool v.1.2 [34]. The digital DNA–DNA hybridization (dDDH) values were obtained using the genome-to-genome distance calculator (GGDC v.3.0) provided by the Leibniz Institute DSMZ (Braunschweig, Germany) [35]. Average amino acid identity (AAI) values were calculated using the ‘aai.rb’ script from the Enveomics collection [32].

In addition, the Enveomics tool was employed to conduct a pan-genome analysis [32]. The pan-genome encompasses three sets of genes: core, variable (shared by some but not all of the strains), and singleton (strain-specific) gene clusters [36]. The visualization of the pan-genome was carried out using the Anvi’o tool [37]. To display the core, variable (shell), and strain-specific genes, a flower plot was generated using the ‘plotrix’ package in R v.3.8.2. Characteristic curves illustrating the evolution of the pan-genome and core-genome of the genus *Halomicroarcula* were represented using the pan-genome profile analysis tool (PanGP) [38]. A heatmap based on the presence and absence of the genes within the shell genome was produced using the “gplot” R package v.3.1.3. To compare the orthologous clusters (OCs) between strains S1CR25-12^T^ and S3CR25-11^T^ and its phylogenomically closest related species, namely, *Halomicroarcula salinisoli* F24A^T^ and *Halomicroarcula laminariae* LYG-108^T^, the OrthoVenn3 online tool was used [39]. Additionally, the isoelectric points of predicted proteins were computed using the ‘iep’ program from the EMBOSS package v.6.5.7.0 [40].

### 2.5. Phenotypic and Chemotaxonomic Characterization

A complete phenotypic characterization was conducted in accordance with the minimal standards for the description of new taxa within the order class *Halobacteria* [41]. Cell morphology and motility were examined by cultivating the strains on 25% R2A liquid medium in a shaking incubator at 200 rpm at 37 °C, and the cells were observed using a light phase-contrast microscope (Zeiss Axioscope 5). Gram staining was performed following the method described by Dussault [42]. To determine the optimal salt requirements, strains S1CR25-12^T^ and S3CR25-11^T^ were cultured in R2A medium with different salt concentrations (0.5%, 5%, 7.5%, 10%, 12%, 15%, 17%, 20%, 22%, 25%, and 30% [*w*/*v*]). Similarly, the pH (5.0, 6.0, 6.5, 7.0, 7.5, 8.0, 8.5, 9.0, and 9.5) and temperature (from 20 °C to 55 °C, at 5 °C intervals, in addition to 37 °C) ranges and optimal values supporting growth were determined in 25% R2A buffered medium. Colonial morphology, pigmentation, and size were examined on 25% R2A solid medium after 10 days of incubation at 37 °C. Anaerobic growth capability was assessed by incubating 25% R2A medium plates supplemented with alternative electron acceptors (L-arginine, DMSO, and KNO_3_) for 14 days at 37 °C in a GasPak system using AnaeroGen (Oxoid, Horsham, UK). The catalase test involved the addition of a few drops of 3% H_2_O_2_ (*v*/*v*) to a young culture of the microorganisms [43]. Oxidase activity was determined using 1% (*v*/*v*) tetramethyl-p-phenylenediamine [44]. Tween 80 hydrolysis was examined as described by Gutiérrez and González [45]. The hydrolyses of starch, gelatin, and casein were detected according to Mata et al. [46]. Aesculin hydrolysis was examined as described by Barrow et al. [47]. H_2_S production was detected using a lead-acetate-impregnated strip [48]. The urease test [49] was carried out to evaluate the ability of the strains to hydrolyze urea. Methyl red and Voges–Proskauer tests were determined according to Oren et al. [41]. The determination of nitrate and nitrite reduction was performed by following the methodology of Gerhardt et al. [50]. The indole test was conducted as described by Kovács [51]. Acid production from carbohydrates and the utilization of carbohydrates, alcohols, organic acids, and amino acids as sole carbon or/and nitrogen sources of energy were determined following the methodology described by Ventosa et al. [52]. Recently, Durán-Viseras [12] conducted a phenotypic characterization in the same laboratory of the following strains: *Halomicroarcula pellucida* CECT 7537^T^, *Halomicroarcula limicola* JCM 18640^T^, *Halomicroarcula nitratireducens* F27^T^, *Halomicroarcula rubra* F13^T^, and *Halomicroarcula salinisoli* F24A^T^, employing the same methodology as that used in our study. Therefore, we used those species as reference strains for comparative phenotypic characterization in our investigation.

The characterization and differentiation of haloarchaea at the genus level have benefited from the analysis of polar lipids, which serve as useful taxonomic markers [41,53]. In this study, the polar lipids were extracted from the cell biomass of the investigated strains, S1CR25-12^T^ and S3CR25-11^T^, as well as from the reference strains *Halobacterium salinarum* DSM 3754^T^, *Halorubrum saccharovorum* DSM 1137^T^, *Halomicroarcula pellucida* CECT 7537^T^, *Halomicroarcula nitratireducens* F27^T^, *Halomicroarcula rubra* F13^T^, and *Halomicroarcula salinisoli* F24A^T^, using a chloroform/methanol extraction system. The identification of polar lipid profiles was accomplished through high-performance thin-layer chromatography (HPTLC) on silica gel glass plates (Merck, Rahway, NJ, USA), employing a chloroform-methanol-90% (*v*/*v*) acetic acid (39.4/2.42/18.18 mL) solvent system [54,55]. The visualization of polar lipids was achieved by applying 5% (*v*/*v*) sulfuric acid followed by heating at 160 °C, while phospholipids were detected using the molybdenum blue spray reagent.

### 2.6. Metagenomic Fragment Recruitment Analyses

Fragment recruitments with seven environmental metagenomic datasets available from databases were carried out to identify and quantify the presence of the eight *Halomicroarcula* species, as well as of strains S1CR25-12^T^ and S3CR25-11^T^ in various saline habitats (Supplementary Table S1). Genomes of *Haloquadratum walsbyi* C23^T^ (GCF_000237865.1), and *Spiribacter salinus* M19-40^T^ (GCF_000319575.2) were included as references for comparison. To minimize analysis bias, contigs from each genome were concatenated, and subsequently, rRNA gene sequences were masked. A Blastn search was executed with specific parameters (alignment length ≥ 50 nt, similarity > 95%, E-value ≤ 10^−5^) to map quality-filtered metagenomic reads against each genome. Recruitment plot representations were generated in R v.4.2.1 using the library “Hmisc” [56].

## 3. Results and Discussion

### 3.1. Physicochemical Features of the Sampling Sites

Two soil samples, designated as 1C and 3C, collected from the hypersaline soils of the Odiel Saltmarshes in Huelva (Spain) were analyzed. The physicochemical properties of sample 1C, from which strain S1CR25-12^T^ was isolated, exhibited a pH of 8.4 and electrical conductivity of 35.7 mS/cm. Sample 3C, corresponding to the isolation place of strain S3CR25-11^T^, displayed a pH value of 7.3 and electrical conductivity of 112.6 mS/cm. The high electrical conductivity indicated that the soils under study can be classified as hypersaline. However, these samples were subjected to analytical testing for the presence of heavy metals (Table 1) because of previous metallurgical and industrial activities in the area. The concentrations of certain prevalent heavy metals (cadmium, copper, and lead) in both soil samples were high, but they were found to conform to the established standards for non-contaminated soils as stipulated by the Environment Department of the regional Government of Andalusia [57]. However, the concentrations of arsenic and zinc in the tested soil samples exceeded the reference intervals, indicating a potential heavy metal tolerance and/or resistance of the two isolated strains, as it is known that some *Halomicroarcula* species possess metal resistance genes against the mentioned heavy metals [12]. Haloarchaeal strains have previously demonstrated significant capabilities in resisting toxic heavy metals [58,59,60,61,62] and further investigations should be pursued, as they exhibit considerable potential for effective implementation in bioremediation strategies.

### 3.2. Unveiling Two Novel Species of the Genus *Halomicroarcula* through Phylogenetic and Phylogenomic Analyses

**Figure 1:**
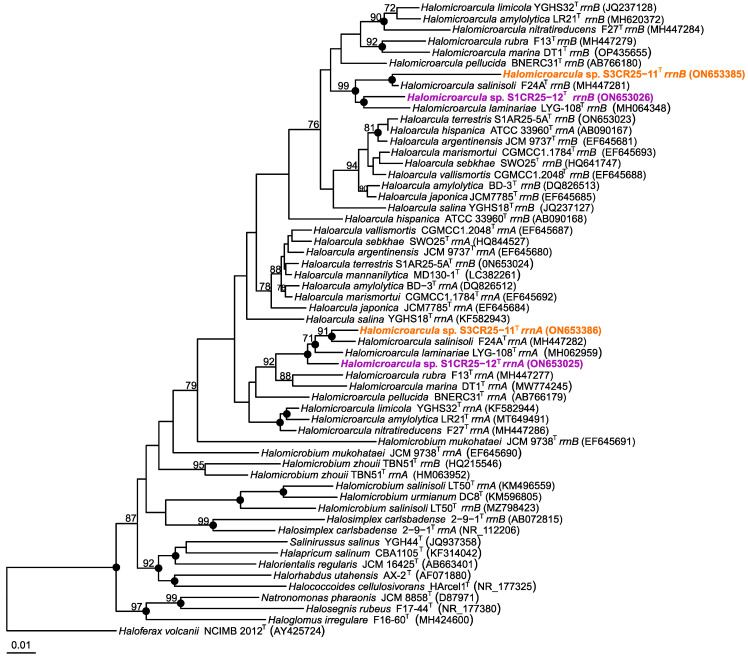
Neighbor-joining phylogenetic tree based on the 16S rRNA gene sequence comparison of strains S1CR25-12^T^ and S3CR25-11^T^, and other related species within the family *Haloarculaceae*. The species *Haloferax volcanii* NCIMB 2012^T^ was used as outgroup. Sequence accession numbers are shown in parentheses. Bootstrap values higher than 70% are shown at branch points. Filled circles indicate branches that were recovered for the trees obtained using the neighbor-joining, maximum-likelihood, and maximum-parsimony algorithms. Bar, 0.01 expected substitutions per nucleotide position.

**Figure 2:**
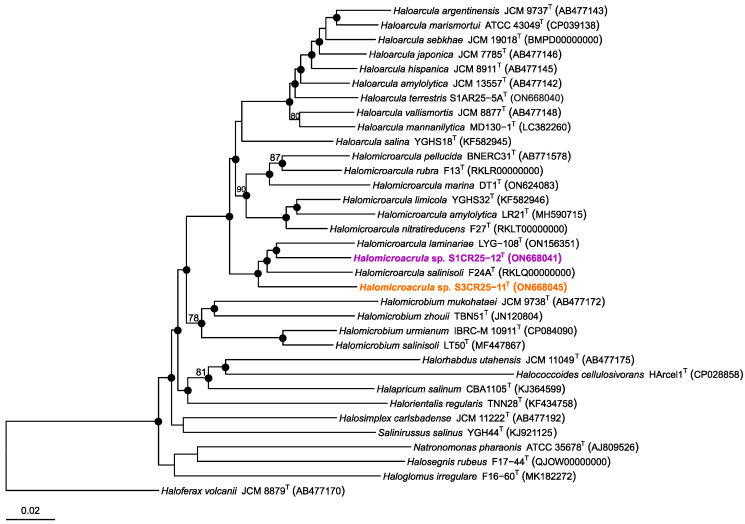
Neighbor-joining phylogenetic reconstruction based on *rpoB’* gene sequences of strains S1CR25-12^T^ and S3CR25-11^T^, and related species of the family *Haloarculaceae*. The species *Haloferax volcanii* JCM 8879^T^ was used as outgroup. Sequence accession numbers are shown in parentheses. Bootstrap values (%) higher than 70% are indicated at branch points. Filled circles indicate that the corresponding nodes were obtained in the trees generated with the neighbor-joining and maximum-likelihood algorithms. Bar, 0.01 expected substitutions per nucleotide position.

Strains S1CR25-12^T^ and S3CR25-11^T^ were isolated as part of an investigation focused on the characterization of prokaryotes from hypersaline soils located in the Odiel Saltmarshes Natural Area in Huelva, Spain. The analysis of the 16S rRNA gene sequences allowed us to determine the initial phylogenetic position of the isolates, which were related to the genus *Halomicroarcula*. Species within the genus *Halomicroarcula* typically possess multiple copies of the 16S rRNA gene with sequence heterogeneity [6,8,9,10,11,12]. In the case of the studied strains, we determined and compared the following 16S rRNA gene sequences with type strains of the *Halomicroarcula* species: *rrnA* 1464 bp, and *rrnB* 1323 bp of strain S1CR25-12^T^, and *rrnA* 1464 bp, and *rrnB* 1463 bp of strain S3CR25-11^T^. Strains S1CR25-12^T^ and S3CR25-11^T^ displayed the highest sequence identity with *Halomicroarcula salinisoli* F24A^T^ (97.9% and 99.1%, respectively) and *Halomicroarcula laminariae* LYG-108^T^ (98.1% and 98.0%, respectively). The presence of multiple copies with sequence variations can pose challenges in classification and may lead to an overestimation of prokaryotic diversity [63,64]. It has been demonstrated that the 16S rRNA gene has limitations as a taxonomic marker in the class *Halobacteria* [65], while the *rpoB’* gene serves as a useful complementary tool to determine the phylogenetic position of new strains within this class [66]. Analysis of the *rpoB’* gene sequences revealed that isolates S1CR25-12^T^ and S3CR25-11 were again most closely related to *Halomicroarcula laminariae* LYG-108^T^ and *Halomicroarcula salinisoli* F24A^T^, showing sequence identities of 94.0–92.7, and 93.5–92.7%, respectively. Phylogenetic reconstructions based on the 16S rRNA gene (Figure 1) and *rpoB’* gene (Figure 2) sequences confirmed the affiliation of isolates S1CR25-12^T^ and S3CR25-11^T^ within the genus *Halomicroarcula*.

The relevant genomic characteristics of strains S1CR25-12^T^, S3CR25-11^T^, and the type strains of the eight species of the genus *Halomicroarcula* are shown in Supplementary Table S2. The maximum-likelihood phylogenomic tree reconstruction, based on the comparison of 157 core-orthologous proteins, revealed that the two new strains formed a cluster with *Halomicroarcula salinisoli* F24A^T^ and *Halomicroarcula laminariae* LYG-108^T^.

**Figure 3:**
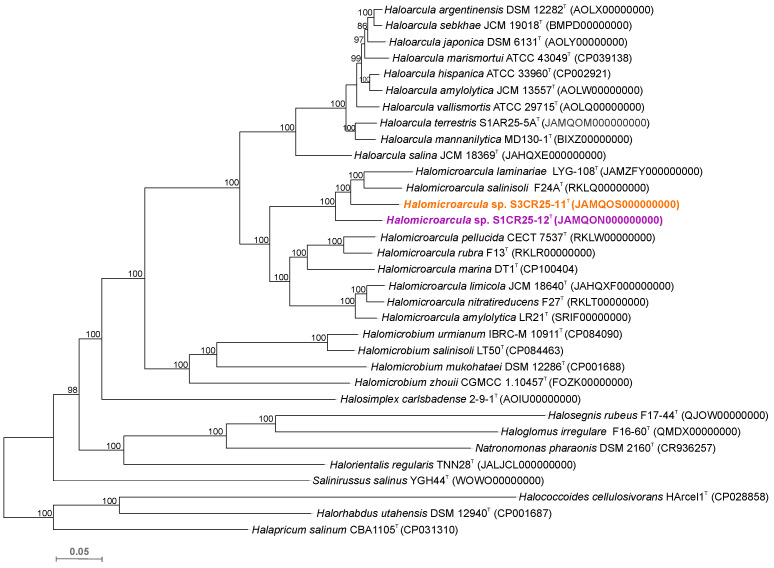
Approximately maximum-likelihood phylogenomic tree based on the comparison of 157 core-orthologous proteins showing the relationships between strains S1CR25-12^T^, S3CR25-11^T^, members of the genus *Halomicroarcula*, and other related species within the family *Haloarculaceae*. Sequence accession numbers are shown in parentheses. Branch support values (%) are computed with the Shimodaira–Hasegawa test and are shown at branch points. Bar, 0.05 substitutions per amino acid position.

**Figure 4:**
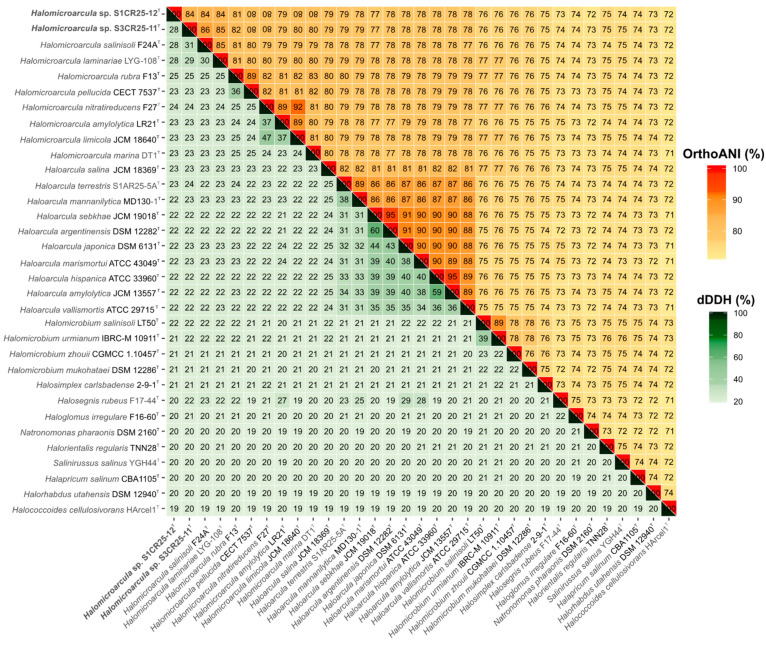
Heatmap displaying orthologous average nucleotide identity (OrthoANI) (upper right) and digital DNA–DNA hybridization (dDDH) (lower left) percentages among strains S1CR25-12^T^ and S3CR25-11^T^, members of the genus *Halomicroarcula*, and other related species of the family *Haloarculaceae*.

**Figure 5:**
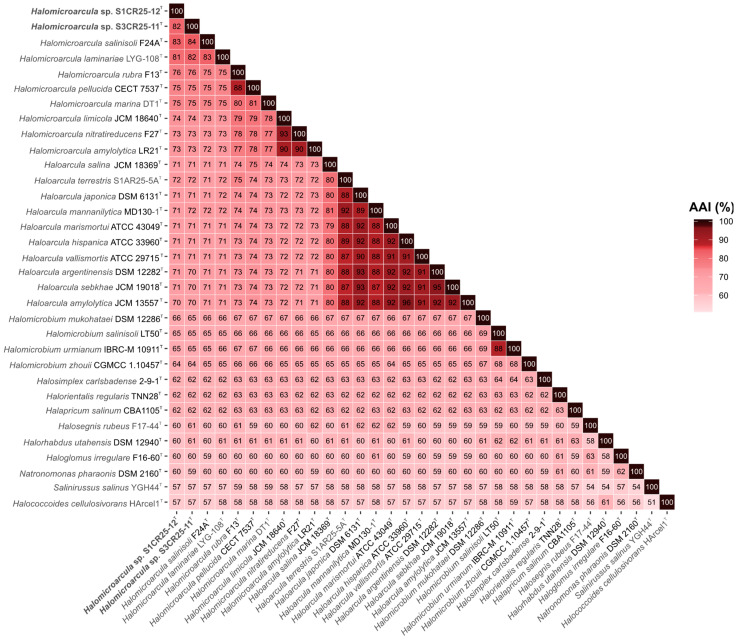
Heatmap showing average amino acid identity (AAI) percentages among *Halomicroarcula* species, including strains S1CR25-12^T^ and S3CR25-11^T^, and other related species of the family *Haloarculaceae*.

However, their phylogenomic divergence suggests that strains S1CR25-12^T^ and S3CR25-11^T^ might constitute two new species within the genus *Halomicroarcula* (Figure 3). To confirm that hypothesis, orthologous average nucleotide identity (OrthoANI) and digital DNA–DNA hybridization (dDDH) were calculated among the studied strains and the type strains of the species of the family *Haloarculaceae*. The comparative analysis of genomic identity revealed that the new isolates exhibited percentages ranging from 86.0 to 78.9% (OrthoANI), 30.9 to 22.7% (dDDH), and 84.2 to 72.6% (AAI) when compared to *Halomicroarcula* species. Specifically, the OrthoANI and dDDH values between strains S1CR25-12^T^ and S3CR25-11^T^ were 84.4% and 28.1%, respectively, unequivocally confirming that these two strains represent distinct species based on established thresholds for prokaryotic species classification [67,68,69,70]. Additionally, the AAI value between the two studied strains was 82.2%, providing further evidence that strains S1CR25-12^T^ and S3CR25-11^T^ are two different species and they should be classified within the genus *Halomicroarcula* (Figure 4 and Figure 5).

### 3.3. Comparative Genomic Analysis Provides Insights into the Genome Structure and the Evolutionary Relationships

**Figure 6:**
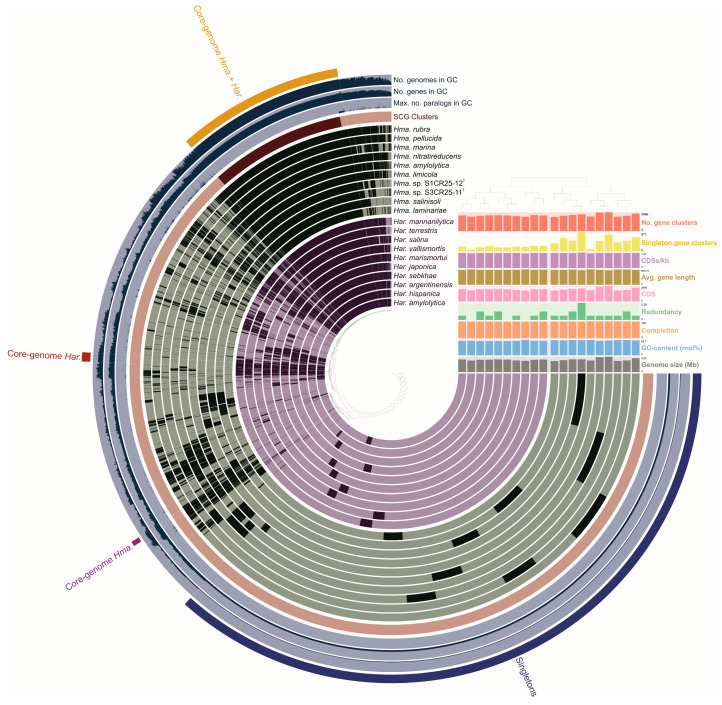
Comparative pan-genome analysis showing the comparison of 20 *Halomicroarcula* and *Haloarcula* type strains: *Hma. rubra* F13^T^, *Hma. pellucida* CECT 7537^T^, *Hma. marina* DT1^T^, *Hma. nitratireducens* F27^T^, *Hma. amylolytica* LR21^T^, *Hma. limicola* JCM 18640^T^, *Hma*. sp. S1CR25-12^T^, *Hma*. sp. S3CR25-11^T^, *Hma. salinisoli* LT50^T^, *Hma. laminariae* LYG-108^T^, *Har. mannanilytica* MD130-1^T^, *Har. terrestris* S1AR25-5A^T^, *Har. salina* JCM 18369^T^, *Har. vallismortis* ATCC 29715^T^, *Har. marismortui* ATCC 43049^T^, *Har. japonica* DSM 6131^T^, *Har. sebkhae* JCM 19018^T^, *Har. argentinensis* DSM 12282^T^, *Har. hispanica* ATCC 33960^T^, and *Har. amylolytica* JCM 13557^T^. In the layers, dark colors indicate the presence of a gene cluster and light colors indicate its absence.

**Figure 7:**
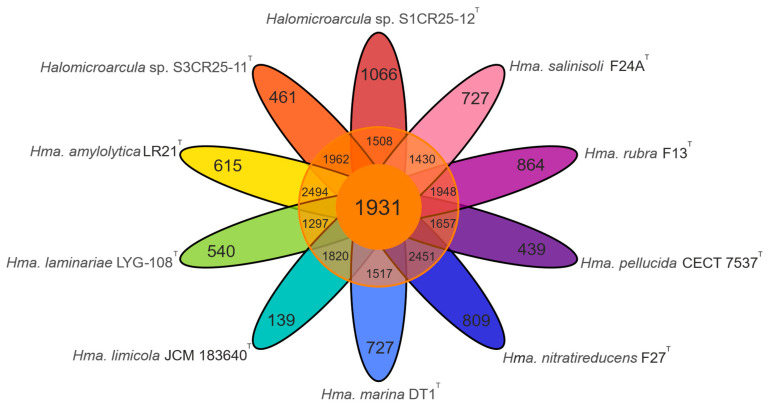
Flower plot showing the core (in the center), variable (in the annulus), and strain-specific (in the petals) genes of ten analyzed *Halomicroarcula* strains.

**Figure 8:**
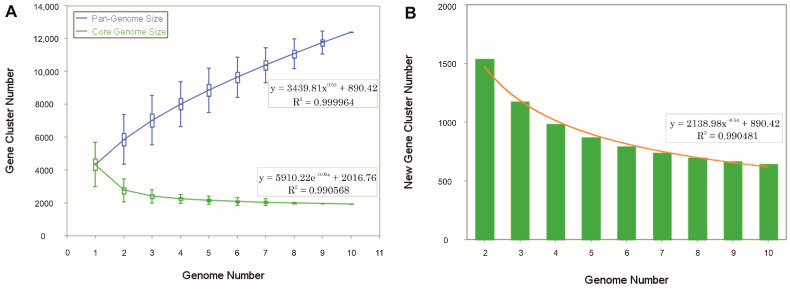
The evolution of the pan-genome and core-genome sizes of species of the genus *Halomicroarcula*. (**A**) Gene accumulation curves of the pan-genome (blue) and the core genome (green), with the curve representing the least squares fit of the power law for average values. (**B**) Number of new genes and fit curve (orange) with an increasing number of *Halomicroarcula* genomes.

**Figure 9:**
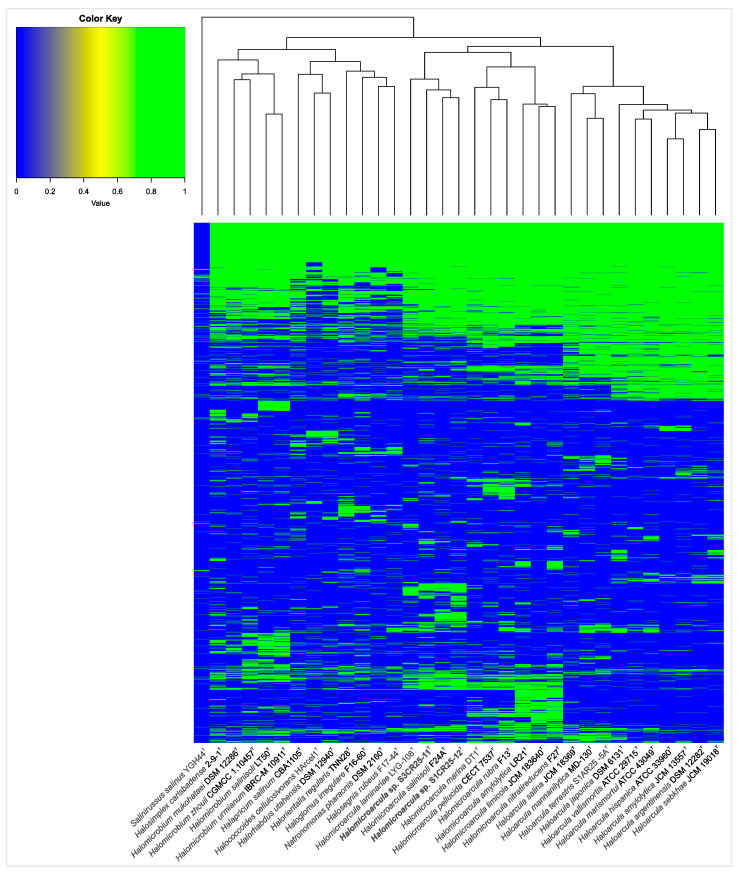
Heatmap based on the presence/absence of the variable gene clusters within the family *Haloarculaceae*. Each row corresponds to a gene cluster, and each column corresponds to a strain. The green color represents the presence of the gene, while the blue color represents its absence.

The pan-genome is characterized as the ensemble of genes that includes genes shared by all members (core genome), as well as genes that are unique to specific individuals or subsets of the group [71]. The 43,781 protein CDSs detected in the 10 analyzed genomes of *Halomicroarcula* were grouped into 5997 orthologous gene clusters (1931 core genes and 4066 variable genes) and 6387 singletons (cloud), with a pan-genome constituted of a total of 12,384 gene clusters (Figure 6 and Figure 7). Pan-genome and core-genome size evolution (Figure 8) describe the changes in the total gene repertoire (pan-genome) and the set of shared genes (core genome) within the genus *Halomicroarcula* over the number of genomes included into the analyzed group. The pan-genome of the genus *Halomicroarcula* is considered open as the addition of new genomes consistently introduces new genes, while the core genome is represented as closed as its size is relatively stable over group size and the set of essential genes remains conserved. A visual representation of a binary matrix that indicates the occurrence (presence or absence) of 9657 variable gene clusters within the family *Haloarculaceae* is shown in Figure 9. The clusters of variable genes are unique for each genus, providing a snapshot of the genomic diversity within a microbial community of the family *Haloarculaceae*. The studied new haloarchaeal isolates, along with the phylogenomically closest species *Halomicroarcula salinisoli* F24A^T^ and *Halomicroarcula laminariae* LYG-108^T^, demonstrated a common set of 2366 orthologous gene clusters (OCs), as shown in Supplementary Figure S2A. Strains S1CR25-12^T^ and S3CR25-11^T^ shared a total of 2972 OCs and possessed 64 and 56 specific orthologous clusters, respectively, confirming that the studied strains represent new species (Supplementary Figure S2B).

Furthermore, the isoelectric points of predicted proteins were computed for all members of the genus *Halomicroarcula* and other reference species. Strains S1CR25-12^T^ and S3CR25-11^T^ displayed a congruent isoelectric profile with the *Halomicroarcula* species, characterized by a peak at around 4 (Supplementary Figure S3). This acidic proteome indicates a “salt-in” osmoregulation strategy [72].

### 3.4. Phenotypic and Chemotaxonomic Characterization Substantiates the Novel Species Statuses and Placement within the Genus *Halomicroarcula*

A comprehensive phenotypic analysis of the two novel strains, S1CR25-12^T^ and S3CR25-11^T^, encompassing morphological, physiological, biochemical, and nutritional characteristics, was conducted. The summarized results of the phenotypic characteristics are shown in Supplementary Table S3, along with the descriptions of the new species.

The HPTLC analysis revealed the lipid composition of strains S1CR25-12^T^ and S3CR25-11^T^, which included phosphatidylglycerol (PG), phosphatidylglycerol phosphate methyl ester (PGP-Me), phosphatidylglycerol sulfate (PGS), and a glycolipid chromatographically identical to sulfated diglycosyl diether (S-DGD-1) (Supplementary Figure S4A,B). These polar lipid profiles correspond to the established polar lipid patterns observed in species of the genus *Halomicroarcula* [6,9,12] validating the taxonomic affiliation of the studied strains within this genus.

### 3.5. Ecological Distribution of the Genus *Halomicroarcula*

**Figure 10:**
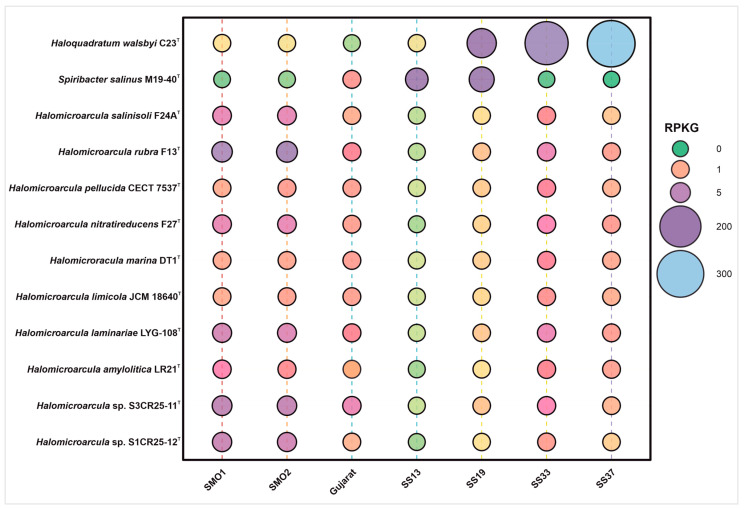
Bubble chart of relative abundances represented as RPKG (reads recruited per kilobase of genome per gigabase of metagenome) of strains S1CR25-12^T^ and S3CR25-11^T^, the species of the genus *Halomicroarcula*, and two reference species, namely, the haloarchaeon *Haloquadratum walsbyi* and the bacterial species *Spiribacter salinus*.

A metagenomic fragment recruitment analysis was carried out to study the distribution and abundance of DNA fragments derived from the two novel species, represented by strains S1CR25-12^T^ and S3CR25-11^T^, and other species of the genus *Halomicroarcula*. Supplementary Figure S5 shows the recruitments of strains S1CR25-12^T^ and S3CR25-11^T^ against seven metagenomic databases from hypersaline soils located in Huelva (SMO1 and SMO2) [73]; four ponds of Santa Pola salterns with different salinities, from 13% to 37% (SS13, SS19, SS33, SS37) [74,75]; and a saline desert soil from Gujarat [76]. The recruitments were abundant, especially in hypersaline soils located in the Odiel Saltmarshes in Huelva. This was mainly represented by the two studied strains S3CR25-11^T^, S1CR25-12^T^, which were isolated from this saline habitat, and *Halomicroarcula rubra* F13^T^, followed by the ponds of the salterns of Santa Pola with salt concentrations of 33% and 37% (*w*/*v*), respectively (Figure 10). Lower recruitment values were detected at the lower salt concentrations, thus confirming the extremely halophilic nature of the species of the genus *Halomicroarcula.*

## 4. Conclusions

In this investigation, focused on the characterization of prokaryotes inhabiting the hypersaline soils of the Odiel Saltmarshes in Huelva (Spain), the haloarchaeal strains S1CR25-12^T^ and S3CR25-11^T^ were isolated. Through the initial analysis of the 16S rRNA gene sequences, the isolates were assigned to the genus *Halomicroarcula*. The existence of divergent copies of the 16S rRNA gene detected in the studied strains is a typical characteristic of the species of this genus. To enhance the taxonomic resolution, we adopted an approach that incorporates comprehensive genome analysis, including OGRI calculations, determination of their phylogenomic positions, and the study of the genomic diversity and evolution. This study, encompassing phylogenetic analysis, polar lipid profiling, phenotypic characterization, and comparative genomic analysis, confirms that strains S1CR25-12^T^ and S3CR25-11^T^ represent novel species within the genus *Halomicroarcula*. Therefore, the names *Halomicroarcula saliterrae* sp. nov. and *Halomicroarcula onubensis* sp. nov. are proposed for strains S1CR25-12^T^ and S3CR25-11^T^, respectively. A detailed description of the two new species is provided below. To conclude, the analysis of metagenomic fragment recruitments indicated the occurrence of *Halomicroarcula* species across diverse hypersaline habitats. The genus exhibited its highest prevalence in the hypersaline soils of the Odiel Saltmarshes, followed by a substantial presence in the hypersaline ponds of the Santa Pola salterns, located in Alicante (Spain), with salt concentrations of 33% and 37% (*w*/*v*), respectively. These findings further affirm the extremely halophilic characteristics associated with the genus *Halomicroarcula*.

**Description of *Halomicroarcula saliterrae* sp. nov.**

*Halomicroarcula saliterrae* (sa.li.ter’rae. L. masc. n. *sal*, salt; L. fem. n. *terra*, land; N.L. gen. n. *saliterrae*, of saline soil).

Cells are motile, Gram-stain-negative, 0.5 × 2–4 μm, pleomorphic rods. Colonies are small, circular, entire, 0.2–0.3 mm in diameter, and red-pigmented after 10 days of incubation at 37 °C. They are extremely halophilic, being capable of growth within salt concentrations ranging from 15 and 30% (*w*/*v*), with optimal growth at 25% (*w*/*v*). They display a pH tolerance range of 6.0–9.0, with an optimum pH of 7.0–8.0, and a temperature range of 20–50 °C, with an optimum temperature of 37 °C. No growth is observed under anaerobic conditions with L-arginine, potassium nitrate, or DMSO. They are catalase positive and oxidase negative. Aesculin is hydrolyzed but does not exhibit hydrolytic activity towards starch, casein, gelatin, or Tween 80. Nitrate is reduced but nitrite is not. H_2_S and indole are not produced. The methyl red test is positive, whereas urease, Voges–Proskauer, and Simmons’ citrate tests are negative. Acid is produced from D-arabinose, D-galactose, D-glucose, D-xylose, glycerol, and maltose, and is not produced from D-fructose, D-trehalose, lactose, mannitol, and sucrose. The following compounds are used as sole carbon and energy sources: D-galactose, maltose, and salicin, whereas citrate, D-arabinose, D-cellobiose, D-fructose, D-glucose, D-ribose, D-sorbitol, D-xylose, fumarate, glycerol, hippurate, lactose, propionate, sucrose, valerate, and xylitol are not. L-alanine, L-arginine, L-cysteine, L-methionine, L-glycine, L-glutamine L-isoleucine, L-serine, and valine are used as sole carbon, nitrogen, and energy sources. The major polar lipids are phosphatidylglycerol (PG), phosphatidylglycerol phosphate methyl ester (PGP-Me), phosphatidylglycerol sulfate (PGS), and a glycolipid chromatographically identical to sulfated diglycosyl diether (S-DGD-1). The DNA G+C content is 65.3 mol%.

The type strain is S1CR25-12^T^ (= CECT 30620^T^ = CCM 9252^T^), isolated from hypersaline soil located in the Odiel Saltmarshes Natural Area, Huelva, Spain.

The GenBank/EMBL/DDBJ accession numbers for the 16S rRNA and *rpoB’* gene sequences of strain S1CR25-12^T^ are ON653025 (*rrnA*), ON653026 (*rrnB*), and ON668041, respectively. The GenBank/EMBL/DDBJ accession number of the whole genome sequence of strain S1CR25-12^T^ is JAMQON000000000.

**Description of *Halomicroarcula onubensis* sp. nov.**

*Halomicroarcula onubensis* (o.nu.ben’sis. L. fem. adj. *onubensis*, of or belonging to Onuba, the ancient Latin name of Huelva, a city in Spain, where the type strain was isolated).

Cells are motile, Gram-stain-negative, 0.5 × 2–5 μm, pleomorphic rods. Colonies are small, circular, entire, 0.2–0.3 mm in diameter, and have an orange–red pigmentation after 10 days of incubation at 37 °C. They are extremely halophilic, being capable of growth within salt concentrations ranging from 12 and 30% (*w*/*v*), with optimal growth at 25% (*w*/*v*). They display a pH tolerance range of 6.0–9.0, with an optimum pH of 7.0–8.0, and a temperature range of 25–55 °C, with an optimum temperature of 37 °C. No growth is observed under anaerobic conditions with L-arginine, potassium nitrate, or DMSO. They are catalase positive and oxidase negative. Aesculin is slightly hydrolyzed, but casein, gelatin, starch, and Tween 80 are not. Nitrate is reduced but nitrite is not. H_2_S and indole are not produced. The methyl red test is positive and urease, Voges–Proskauer, and Simmons’ citrate tests are negative. Acid is produced from D-arabinose, D-fructose, D-glucose, D-xylose, glycerol, mannitol, and sucrose and is not produced from D-galactose, D-trehalose, lactose, and maltose. The following compounds are used as sole carbon and energy sources: D-glucose, D-xylose, fumarate, hippurate, sucrose, and xylitol, whereas citrate, D-arabinose, D-galactose, D-cellobiose, D-fructose, D-ribose, D-sorbitol, glycerol, lactose, maltose, propionate, salicin, and valerate are not. L-cysteine and L-serine are used as sole carbon, nitrogen, and energy sources, whereas L-alanine, L-arginine, L-methionine, L-glycine, L-glutamine, L-isoleucine, and valine are not. The major polar lipids are phosphatidylglycerol (PG), phosphatidylglycerol phosphate methyl ester (PGP-Me), phosphatidylglycerol sulfate (PGS), and a glycolipid chromatographically identical to sulfated diglycosyl diether (S-DGD-1). The DNA G+C content is 65.7 mol%.

The type strain is S3CR25-11^T^ (= CECT 30621^T^ = CCM 9254^T^), isolated from hypersaline soil located in the Odiel Saltmarshes Natural Area, Huelva, Spain.

The GenBank/EMBL/DDBJ accession numbers for the 16S rRNA and *rpoB’* gene sequences of strain S3CR25-11^T^ are ON653386 (*rrnA*), ON653385 (*rrnB*), and ON668045, respectively. The GenBank/EMBL/DDBJ accession number of the whole genome sequence of strain S3CR25-11^T^ is JAMQOS000000000.

## Figures and Tables

**Table 1:** Concentrations of the most abundant heavy metals present in the two soil samples (1C and 3C) and reference ranges for non-contaminated soils.

Heavy Metal	1C (mg/kg)	3C (mg/kg)	* Range for Non-Contaminated Soils (mg/kg)
Arsenic	11.1	9.8	2–5
Cadmium	0.5	0.6	0.4–0.8
Copper	85.7	88.0	17–100
Lead	28.8	22.5	10–50
Zinc	84.4	82.5	10–70

* The reference ranges stand for non-contaminated soils in Andalucia (Spain) [57], where the Odiel Saltmarshes National Area is located. Values higher than the reference ranges for non-contaminated soils are highlighted in red.

## Data Availability

All relevant data can be found within the article and Supplementary Materials. Datasets presented in this study can be found in online repositories. The names of the repositories and accession numbers can be found below and can be accessed via https://www.ncbi.nlm.nih.gov/genbank/. The 16S rRNA, *rpoB’* genes, and the genome sequences generated for this study can be found in the GenBank/EMBL/DDBJ database under the accession numbers: ON653025, ON653026, ON653386, ON653385, ON668041, ON668045, JAMQON000000000, and JAMQOS000000000.

## Supplementary Materials

The following supporting information can be downloaded at: https://www.mdpi.com/article/10.3390/microorganisms12020334/s1. Table S1: Data related to the metagenomic datasets used for the fragment recruitment analyses. Table S2: Main genomic features of strains S1CR25-12^T^, S3CR25-11^T^, and the type strains of species of the genus *Halomicroarcula.* Table S3: Differential characteristics between strains S1CR25-12^T^ and S3CR25-11^T^, and related species of the genus *Halomicroarcula*. Figure S1: Isolation places designated as 1C and 3C (A) and hypersaline soil located in Odiel Saltmarshes (Huelva, Spain) where studied strains S1CR25-12^T^ and S3CR25-11^T^ were isolated (B). Figure S2: Venn diagrams showing the distribution of the orthologous gene clusters (OCs) determined for strains S1CR25-12^T^, S3CR25-11^T^, and their phylogenomically closest related species, *Halomicroarcula salinisoli* F24A^T^ and *Halomicroarcula laminariae* LYG-108^T^ (A), and the distribution of the orthologous gene clusters (OCs) between the two studied strains S1CR25-12^T^ and S3CR25-11^T^, respectively (B). Figure S3: Isoelectric point comparison of predicted proteins from the type strains of species of the genus *Halomicroarcula*, including the new isolates S1CR25-12^T^ and S3CR25-11^T^, and other reference strains. Figure S4: High-performance thin-layer chromatography (HPTLC) showing the comparison of the polar lipid (A) and phospholipid (B) profiles between strains S1CR25-12^T^, S3CR25-11^T^, and other *Halomicroarcula* species, as well as *Halorubrum saccharovorum* and *Halobacterium salinarum*. Figure S5: Recruitment plots of the two novel strains S1CR25-12^T^ (A) and S3CR25-11^T^ (B) against seven different hypersaline metagenomic datasets.

## References

[1] Galisteo C., de la Haba R.R., Sánchez-Porro C., Ventosa A. (2023). A step into the rare biosphere: Genomic features of the new genus *Terrihalobacillus* and the new species *Aquibacillus salsiterrae* from hypersaline soils. Front. Microbiol..

[2] Rodríguez-Valera F., Rodríguez-Valera F. (1988). Characteristics and microbial ecology of hypersaline environments. Halophilic Bacteria.

[3] Ventosa A., Logan N.A., Lappin-Scott H.M., Oyston. P.C.E. (2006). Unusual micro-organisms from unusual habitats: Hypersaline environments. Prokaryotic Diversity: Mechanisms and Significance.

[4] Sainz A., Grande J.A., de la Torre M.L., Sánchez-Rodas D. (2002). Characterization of sequential leachate discharges of mining waste rock dumps in the Tinto and Odiel rivers. J. Environ. Manag..

[5] Sainz A., Grande J.A., de la Torre M.L. (2004). Characterization of heavy metal discharge into the Ria of Huelva. Environ. Int..

[6] Echigo A., Minegishi H., Shimane Y., Kamekura M., Itoh T., Usami R. (2013). *Halomicroarcula pellucida* gen. nov., sp. nov., a non-pigmented, transparent-colony-forming, halophilic archaeon isolated from solar salt. Int. J. Syst. Evol. Microbiol..

[7] Parte A.C., Sardà Carbasse J., Meier-Kolthoff J.P., Reimer L.C., Göker M. (2020). List of Prokaryotic names with Standing in Nomenclature (LPSN) moves to the DSMZ. Int. J. Syst. Evol. Microbiol..

[8] Ma X., Hu Y., Li X.X., Tan S., Cheng M., Hou J., Cui H.L. (2023). *Halomicroarcula laminariae* sp. nov. and *Halomicroarcula marina* sp. nov., extremely halophilic archaea isolated from salted brown alga *Laminaria* and coastal saline-alkali lands. Int. J. Syst. Evol. Microbiol..

[9] Zhang W., Cui H. (2014). *Halomicroarcula limicola* sp. nov., isolated from a marine solar saltern, and emended description of the genus *Halomicroarcula*. Int. J. Syst. Evol. Microbiol..

[10] Zhang W., Cui H. (2015). *Halomicroarcula salina* sp. nov., isolated from a marine solar saltern. Int. J. Syst. Evol. Microbiol..

[11] Chen F., Xu Y., Sun S., Shi X., Liu A., Chen S. (2020). *Halomicroarcula amylolytica* sp. nov., a novel halophilic archaeon isolated from a salt mine. Int. J. Syst. Evol. Microbiol..

[12] Durán-Viseras A., Sánchez-Porro C., Ventosa A. (2021). Genomic insights into new species of the genus *Halomicroarcula* reveals potential for new osmoadaptative strategies in halophilic archaea. Front. Microbiol..

[13] Subov N.N. (1931). Oceanographical Tables.

[14] Marmur J. (1961). A procedure for the isolation of deoxyribonucleic acid from micro-organisms. J. Mol. Biol..

[15] DeLong E.F. (1992). Archaea in coastal marine environments. Proc. Natl. Acad. Sci. USA.

[16] Arahal D.R., Dewhirst F.E., Paster B.J., Volcani B.E., Ventosa A. (1996). Phylogenetic analyses of some extremely halophilic archaea isolated from Dead Sea water, determined on the basis of their 16S rRNA sequences. Appl. Environ. Microbiol..

[17] Fullmer M.S., Soucy S.M., Swithers K.S., Makkay A.M., Wheeler R., Ventosa A., Gogarten J.P., Papke R.T. (2014). Population and genomic analysis of the genus *Halorubrum*. Front. Microbiol..

[18] García-Roldán A., Durán-Viseras A., de la Haba R.R., Corral P., Sánchez-Porro C., Ventosa A. (2023). Genomic-based phylogenetic and metabolic analyses of the genus *Natronomonas*, and description of *Natronomonas aquatica* sp. nov. Front. Microbiol..

[19] Yoon S.H., Ha S.M., Kwon S., Lim J., Kim Y., Seo H., Chun J. (2017). Introducing EzBioCloud: A taxonomically united database of 16S rRNA gene sequences and whole-genome assemblies. Int. J. Syst. Evol. Microbiol..

[20] Altschul S.F., Gish W., Miller W., Myers E.W., Lipman D.J. (1990). Basic local alignment search tool. J. Mol. Biol..

[21] Alzohairy A.M. (2011). BioEdit: An important software for molecular biology. GERF Bull. Biosci..

[22] Ludwig W., Strunk O., Westram R., Richter L., Meier H., Yadhukumar A., Buchner A., Lai T., Steppi S., Jobb G. (2004). ARB: A software environment for sequence data. Nucleic Acids Res..

[23] Felsenstein J. (1981). Evolutionary trees from DNA sequences: A maximum likelihood approach. J. Mol. Evol..

[24] Saitou N., Nei M. (1987). The neighbor-joining method: A new method for reconstructing phylogenetic trees. Mol. Biol. Evol..

[25] Felsenstein J. (1983). Parsimony in systematics: Biological and statistical issues. Annu. Rev. Ecol. Syst..

[26] Galisteo C. (2022). Gitana: Phylogenetic Imaging Tool for Adjusting Nodes and other Arrangements. https://github.com/cristinagalisteo/gitana.

[27] Felsenstein J. (1985). Confidence limits on phylogenies: An approach using the bootstrap. Evolution.

[28] Chun J., Oren A., Ventosa A., Christensen H., Arahal D.R., da Costa M.S., Rooney A.P., Yi H., Xu X.W., De Meyer S. (2018). Proposed minimal standards for the use of genome data for the taxonomy of prokaryotes. Int. J. Syst. Evol. Microbiol..

[29] Prjibelski A., Antipov D., Meleshko D., Lapidus A., Korobeynikov A. (2020). Using SPAdes de novo assembler. Curr. Protoc. Bioinform..

[30] Parks D.H., Imelfort M., Skennerton C., Hugenholtz P., Tyson G.W. (2015). CheckM: Assessing the quality of microbial genomes recovered from isolates, single cells, and metagenomes. Genome Res..

[31] Seemann T. (2014). Prokka: Rapid prokaryotic genome annotation. Bioinformatics.

[32] Rodríguez-R L.M., Konstantinidis K.T. (2016). The Enveomics collection: A toolbox for specialized analyses of microbial genomes and metagenomes. PeerJ.

[33] Price M.N., Dehal P.S., Arkin A.P. (2010). FastTree 2–Approximately maximum-likelihood trees for large alignments. PLoS ONE.

[34] Lee I., Kim Y.O., Park S.C., Chun J. (2016). OrthoANI: An improved algorithm and software for calculating average nucleotide identity. Int. J. Syst. Evol. Microbiol..

[35] Meier-Kolthoff J.P., Sardà Carbasse J., Peinado-Olarte R.L., Göker M. (2022). TYGS and LPSN: A database tandem for fast and reliable genome-based classification and nomenclature of prokaryotes. Nucleic Acids Res..

[36] Medini D., Donati C., Tettelin H., Masignani V., Rappuoli R. (2005). The microbial pan-genome. Curr. Opin. Genet. Dev..

[37] Eren A.M., Esen Ö.C., Quince C., Vineis J.H., Morrison H.G., Sogin M.L., Delmont T.O. (2015). Anvi’o: An advanced analysis and visualization platform for ’omics data. PeerJ.

[38] Zhao Y., Jia X., Yang J., Ling Y., Zhang Z., Yu J., Wu J., Xiao J. (2014). PanGP: A tool for quickly analyzing bacterial pan-genome profile. Bioinformatics.

[39] Sun J., Lu F., Luo Y., Bie L., Xu L., Wang Y. (2023). OrthoVenn3: An integrated platform for exploring and visualizing orthologous data across genomes. Nucleic Acids Res..

[40] Rice P., Longden L., Bleasby A. (2000). EMBOSS: The European Molecular Biology Open Software Suite. Trends Genet..

[41] Oren A., Ventosa A., Grant W.D. (1997). Proposed minimal standards for description of new taxa in the order *Halobacteriales*. Int. J. Syst. Bacteriol..

[42] Dussault H.P. (1955). An improved technique for staining red halophilic bacteria. J. Bacteriol..

[43] Cowan S.T., Steel K.J. (1993). Manual for the Identification of Medical Bacteria.

[44] Kovács N. (1956). Identification of *Pseudomonas pyocyanea* by the oxidase reaction. Nature.

[45] Gutiérrez C., González C. (1972). Method for simultaneous detection of proteinase and esterase activities in extremely halophilic bacteria. Appl. Microbiol..

[46] Mata J.A., Martínez-Cánovas J., Quesada E., Béjar V. (2002). A detailed phenotypic characterisation of the type strains of *Halomonas* species. Syst. Appl. Microbiol..

[47] Barrow G.I., Feltham R.K.A. (2003). Cowan and Steel’s Manual for the Identification of Medical Bacteria.

[48] Clarke P.H. (1953). Hydrogen sulphide production by bacteria. J. Gen. Microbiol..

[49] Christensen W.B. (1946). Urea decomposition as a means of differentiating *Proteus* and paracolon cultures from each other and from *Salmonella* and *Shigella* types. J. Bacteriol..

[50] Gerhardt P., Murray R.G., Wood W.A., Krieg N. (1994). Methods for General and Molecular Bacteriology.

[51] Kovács N. (1928). Eine vereinfachte methode zum nachweis der indolbildung durch bakterien. Z. Immunitätsforsch.

[52] Ventosa A., Quesada E., Rodriguez-Valera F., Ruiz-Berraquero F., Ramos-Cormenzana A. (1982). Numerical taxonomy of moderately halophilic Gram-negative rods. Microbiology.

[53] Torreblanca M., Rodriguez-Valera F., Juez G., Ventosa A., Kamekura M., Kates M. (1986). Classification of non-alkaliphilic halobacteria based on numerical taxonomy and polar lipid composition, and description of *Haloarcula* gen. nov. and *Haloferax* gen. nov. Syst. Appl. Microbiol..

[54] Angelini R., Corral P., Lopalco P., Ventosa A., Corcelli A. (2012). Novel ether lipid cardiolipins in archaeal membranes of extreme haloalkaliphiles. Biochim. Biophys. Acta.

[55] Corral P., Gutiérrez M.C., Castillo A.M., Domínguez M., Lopalco P., Corcelli A., Ventosa A. (2013). *Natronococcus roseus* sp. nov., a haloalkaliphilic archaeon from a hypersaline lake. Int. J. Syst. Evol. Microbiol..

[56] Harrell F., Dupont C. (2022). Hmisc: Harrell Miscellaneous. R Package Version 4.2-0. https://CRAN.R-project.org/package=Hmisc.

[57] (1999). Consejería de Medio Ambiente de la Junta de Andalucía. Los Criterios y Estándares para Declarar un Suelo Contaminado en Andalucía y la Metodología y Técnicas de Toma de Muestra y Análisis para su Investigación. Sevilla: Junta de Andalucía. https://www.juntadeandalucia.es/medioambiente/web/Bloques_Tematicos/Estado_Y_Calidad_De_Los_Recursos_Naturales/Suelo/Criterios_pdf/Presentacion.pdf.

[58] Li X., Meng D., Li J., Yin H., Liu H., Liu X., Cheng C., Xiao Y., Liu Z., Yan M. (2017). Response of soil microbial communities and microbial interactions to long-term heavy metal contamination. Environ. Pollut..

[59] Krzmarzick M.J., Taylor D.K., Fu X., McCutchan A.L. (2018). Diversity and niche of archaea in bioremediation. Archaea.

[60] Vera-Bernal M., Martínez-Espinosa R.M. (2021). Insights on cadmium removal by bioremediation: The case of haloarchaea. Microbiol. Res..

[61] Zhang M., Zhang T., Zhou L., Lou W., Zeng W., Liu T., Yin H., Liu H., Liu X., Mathivanan K. (2022). Soil microbial community assembly model in response to heavy metal pollution. Environ. Res..

[62] Tavoosi N., Akhavan Sepahi A., Amoozegar M.A., Kiarostami V. (2023). Toxic heavy metal/oxyanion tolerance in haloarchaea from some saline and hypersaline ecosystems. J. Basic. Microbiol..

[63] Sun D.L., Jiang X., Wu Q.L., Zhou N.Y. (2013). Intragenomic heterogeneity of 16S rRNA genes causes overestimation of prokaryotic diversity. Appl. Environ. Microbiol..

[64] Ibal J.C., Pham H.Q., Park C.E., Shin J.H. (2019). Information about variations in multiple copies of bacterial 16S rRNA genes may aid in species identification. PLoS ONE.

[65] de la Haba R.R., Corral P., Sánchez-Porro C., Infante-Domínguez C., Makkay A.M., Amoozegar M.A., Ventosa A., Papke R.T. (2018). Genotypic and lipid analyses of strains from the archaeal genus *Halorubrum* reveal insights into their taxonomy, divergence, and population structure. Front. Microbiol..

[66] Minegishi H., Kamekura M., Itoh T., Echigo A., Usami R., Hashimoto T. (2010). Further refinement of the phylogeny of the *Halobacteriaceae* based on the full-length RNA polymerase subunit B’ (*rpoB’*) gene. Int. J. Syst. Evol. Microbiol..

[67] Goris J., Konstantinidis K.T., Klappenbach J.A., Coenye T., Vandamme P., Tiedje J.M. (2007). DNA-DNA hybridization values and their relationship to whole-genome sequence similarities. Int. J. Syst. Evol. Microbiol..

[68] Richter M., Rossello-Mora R. (2009). Shifting the genomic gold standard for the prokaryotic species definition. Proc. Natl. Acad. Sci. USA.

[69] Auch A.F., von Jan M., Klenk H.-P., Göker M. (2010). Digital DNA-DNA hybridization for microbial species delineation by means of genome-to-genome sequence comparison. Stand. Genom. Sci..

[70] Chun J., Rainey F.A. (2014). Integrating genomics into the taxonomy and systematics of the *Bacteria* and *Archaea*. Int. J. Syst. Evol. Microbiol..

[71] Tettelin H., Masignani V., Cieslewicz M.J., Donati C., Medini D., Ward N.L., Angiuoli S.V., Crabtree J., Jones A.L., Durkin A.S. (2005). Genome analysis of multiple pathogenic isolates of *Streptococcus agalactiae*: Implications for the microbial pan-genome. Proc. Natl. Acad. Sci. USA.

[72] Becker E.A., Seitzer P.M., Tritt A., Larsen D., Krusor M., Yao A.I., Wu D., Madern D., Eisen J.A., Darling A.E. (2014). Phylogenetically driven sequencing of extremely halophilic archaea reveals strategies for static and dynamic osmo-response. PLoS Genet..

[73] Vera-Gargallo B., Navarro-Sampedro L., Carballo M., Ventosa A. (2018). Metagenome sequencing of prokaryotic microbiota from two hypersaline soils of the Odiel Salt Marshes in Huelva, Southwestern Spain. Genome Announc..

[74] Fernández A.B., Ghai R., Martin-Cuadrado A.-B., Sánchez-Porro C., Rodriguez-Valera F., Ventosa A. (2014). Prokaryotic taxonomic and metabolic diversity of an intermediate salinity hypersaline habitat assessed by metagenomics. FEMS Microbiol. Ecol..

[75] Ghai R., Pašić L., Fernández A.B., Martin-Cuadrado A.B., Mizuno C.M., McMahon K.D., Papke R.T., Stepanauskas R., Rodriguez-Brito B., Rohwer F. (2011). New abundant microbial groups in aquatic hypersaline environments. Sci. Rep..

[76] Patel R., Mevada V., Prajapati D., Dudhagara P., Koringa P., Joshi C.G. (2015). Metagenomic sequence of saline desert microbiota from wild ass sanctuary, Little Rann of Kutch, Gujarat, India. Genom. Data.

