# Supplementary material for: Decoding the Genomic Profile of the Halomicroarcula Genus: Comparative Analysis and Characterization of Two Novel Species
Source: Microorganisms. 2024 Feb 5;12(2):334. doi: 10.3390/microorganisms12020334 (PMC10892550; doi:10.3390/microorganisms12020334)
Supplement: Supplementary file 1 [file microorganisms-12-00334-s001.zip › microorganisms-2824328-supplementary.pdf]

Supplementary Material

Decoding the Genomic Profile of the *Halomicroarcula* Genus: Comparative Analysis and Characterization of Two Novel Species

Dáša Straková [1], Cristina Sánchez-Porro [1], Rafael R. de la Haba [1] and Antonio Ventosa [1,*]

[1] Department of Microbiology and Parasitology, Faculty of Pharmacy, University of Sevilla, 41012 Sevilla, Spain; daska.strakova@gmail.com (D.S.); sanpor@us.es (C.S.P.); rrh@us.es (R.R.H.); ventosa@us.es (A.V.)

* Correspondence: ventosa@us.es

Supplementary Table S1. Data related to the metagenomic datasets used for the fragment recruitment analyses. NA, not available.

Metagenomic dataset	Sample	Salt concentration	Accession number	Reference
SMO1	Hypersaline soil (Huelva, Spain)	24.0 dS/m	SRR5753725	[73]
SMO2	Hypersaline soil (Huelva, Spain)	54.4 dS/m	SRR5753724	[73]
SS13	Saltern pond (Alicante, Spain)	13 % (w/v)	SRR944625	[74]
SS19	Saltern pond (Alicante, Spain)	19 % (w/v)	SRR328982	[75]
SS33	Saltern pond (Alicante, Spain)	33 % (w/v)	SRR979792	[74]
SS37	Saltern pond (Alicante, Spain)	37 % (w/v)	SRR328983	[75]
Gujarat	Saline desert (Kutch, India)	NA	ERP005612	[76]

Supplementary Table S2. Main genomic features of strains S1CR25-12^{T}, S3CR25-11^{T}, and the type strains of species of the genus *Halomicroarcula*.

**Genomic feature**	**Strain S1CR25-12^{T}**	**Strain S3CR25-11^{T}**	***Hma. pellucida* CECT 7537^{T}**	***Hma. amylolytica* LR21^{T}**	***Hma. laminariae* LYG-108^{T}**	***Hma. limicola* JCM 18640^{T}**	***Hma. marina* DT1^{T}**	***Hma. nitratireducens* F27^{T}**	***Hma. rubra* F13^{T}**	***Hma. salinisoli* F24A^{T}**
Size (Mb)	4.2	4.3	3.9	5.0	3.7	3.9	3.7	5.2	4.7	4.0
Contigs	20	14	8	34	9	5	3	63	35	9
N50 (bp)	650,850	514,788	1,010,699	1,435,272	2,245,722	1,379,950	3,400,000	450,140	308,161	1,343,505
Completeness (%)	99.5	99.1	99.5	99.5	99.5	99.1	99.9	99.5	99.5	99.5
Genome coverage	410X	438X	106X	100X	300X	463X	380X	467X	399X	424X
CDS	4,505	4,354	4,027	5,040	3,768	3,890	4,175	5,191	4,743	4,088
rRNA	5	6	3	4	4	4	9	3	6	5
tRNA	67	77	49	46	46	54	47	54	51	48
G+C (mol%)	65.3	65.7	65.5	62.0	65.0	65.9	65.1	63.2	64.4	64.1
Accession number	JAMQON000000000	JAMQOS000000000	RKLW00000000	SRIF00000000	JAMZFY000000000	JAHQXF000000000	CP100404<br>CP100405<br>CP100406	RKLT00000000	RKLR00000000	RKLQ00000000

Supplementary Table S3. Differential characteristics between strains S1CR25-12^{T} and S3CR25-11^{T}, and related species of the genus *Halomicroarcula.* All data are from this study unless otherwise indicated. +, positive; -, negative; w, weakly positive; ND, not determined. [a]Data from Ma *et al*. [8]. Data of *Halomicroarcula pellucida* CECT 7537^{T}, *Halomicroarcula limicola* JCM 18640^{T}, *Halomicroarcula nitratireducens* F27^{T}, *Halomicroarcula rubra* F13^{T}, and *Halomicroarcula salinisoli* F24A^{T} were obtained from Durán-Viseras *et al.* [12].

**Characteristic**	**Strain S1CR25-12^{T}**	**Strain S3CR25-11^{T}**	***Hma. pellucida* CECT 7537^{T}**	***Hma. laminariae* LYG-108^{T}**	***Hma. limicola* JCM 18640^{T}**	***Hma. nitratireducens* F27^{T}**	***Hma. rubra* F13^{T}**	***Hma. salinisoli* F24A^{T}**
Morphology	Pleomorphic rods	Pleomorphic rods	Pleomorphic	Rods[a]	Pleomorphic	Rods	Rods	Rods
Colony pigmentation	Red	Orange-red	Transparent (non-pigmented)[a]	Red[a]	Red	Orange-red	Red	Pink
NaCl requirement:								
Range (%, w/v)	15–30	12–30	20–30	8–28[a]	10–30	10–30	10–30	15–30
Optimum (%, w/v)	25	25	25	15[a]	25	25–30	30	25
Temperature requirement:								
Range (°C)	20–50	25–55	25–55	20–50[a]	20–50	20–50	25–50	25–50
Optimum (°C)	37	37	40	40[a]	37	37	37	37
pH requirement:								
Range	6.0–9.0	6.0–9.0	6.0–8.5	5.0–9.5[a]	6.0–8.5	6–9.0	6–9.0	6.0–8.5
Optimum	7.0–8.0	7.0-8.0	7.0	7.0[a]	7.5	7.5	7.5–8.0	7.5
Anaerobic growth with:								
DMSO	-	-	ND	-[a]	-	-	-	-
L-Arginine	-	-	ND	-[a]	+	-	-	-
Potassium nitrate	-	-	+	-[a]	-	-	-	-
Hydrolysis of:								
Aesculin	+	w	+	ND	+	-	-	+
Gelatin	-	-	+	-[a]	-	-	+	+
Starch	-	-	-	-[a]	-	+	-	-
Tween 80	-	-	-	-[a]	-	+	-	+
Production of acid from carbohydrates:								
D-Glucose	+	+	+	+[a]	+	+	+	-
D-Mannitol	-	+	+	ND	-	-	-	-
Utilization as sole carbon, (nitrogen), and energy source:								
Citrate	-	-	-	-[a]	-	-	-	+
D-Cellobiose	-	-	-	ND	+	-	-	+
D-Galactose	+	-	-	+[a]	+	+	-	-
D-Glucose	-	+	-	+[a]	+	+	-	+
D-Ribose	-	-	+	-[a]	+	+	-	-
D-Sorbitol	-	-	-	+[a]	-	+	-	+
Fumarate	-	+	-	+[a]	-	+	-	+
Glycerol	-	-	-	+[a]	+	+	-	-
L-Arginine	+	-	-	-[a]	+	-	+	-
L-Cysteine	+	+	+	ND	-	-	-	-
L-Isoleucine	+	-	+	ND	ND	-	-	-
L-Methionine	+	-	-	ND	-	-	+	-
Propionate	-	-	+	ND	+	-	-	-
Salicin	+	-	-	ND	-	+	-	-
Valine	+	-	+	ND	+	-	-	-

A

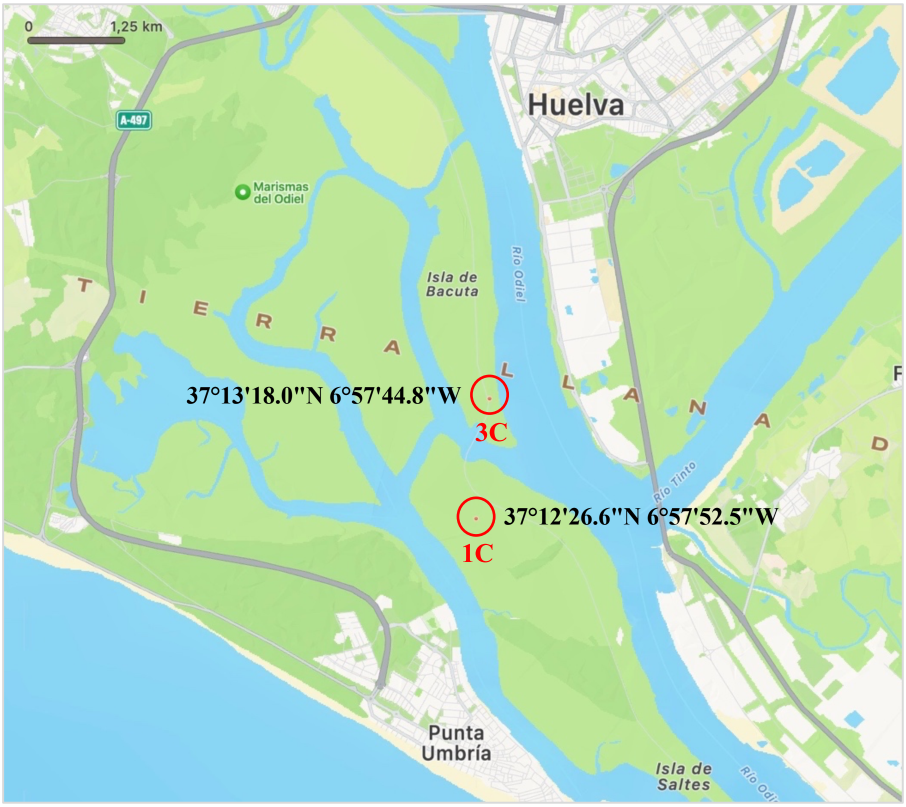

B

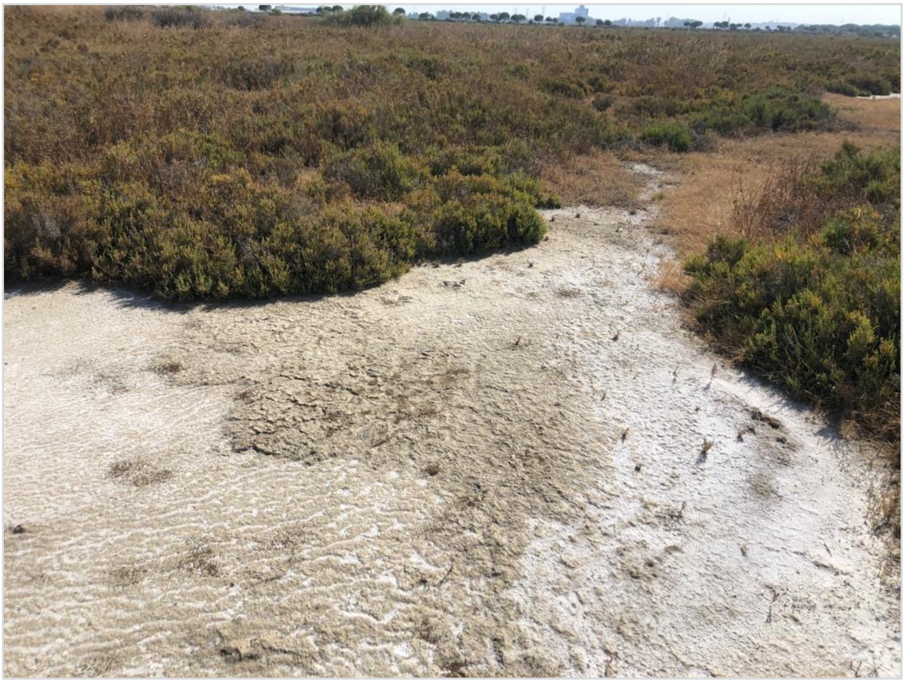

Supplementary Figure S1. Isolation places designated as 1C and 3C (A) and hypersaline soil located in Odiel Saltmarshes (Huelva, Spain) where studied strains S1CR25-12^{T} and S3CR25-11^{T} were isolated (B).

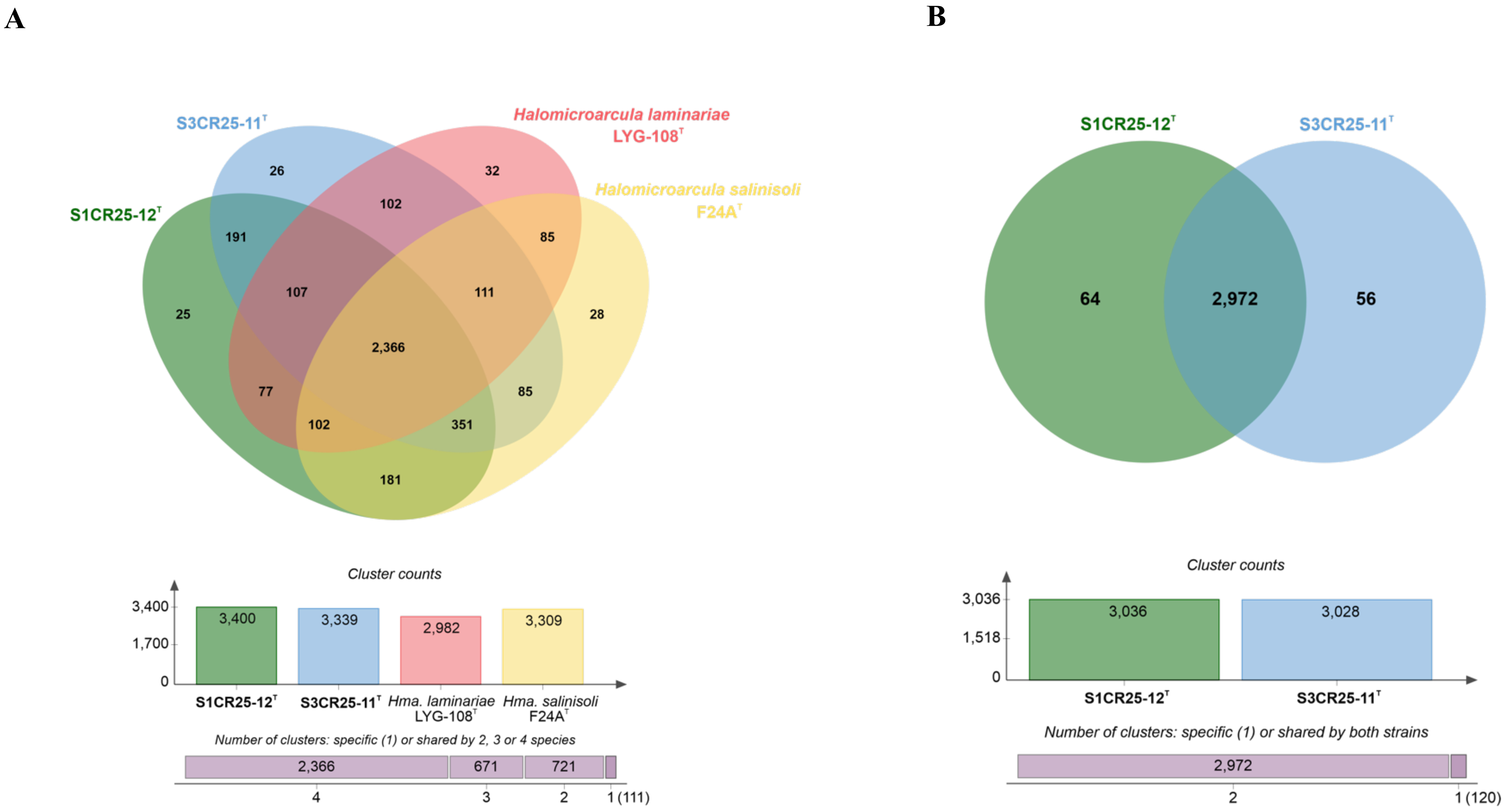

Supplementary Figure S2. Venn diagrams showing the distribution of the orthologous gene clusters (OCs) determined for strains S1CR25-12^T, S3CR25-11^T, and their phylogenomically closest related species, *Halomicroarcula salinisoli* F24A^T and *Halomicroarcula laminariae* LYG-108^T (A), and the distribution of the orthologous gene clusters (OCs) between two studied strains S1CR25-12^T and S3CR25-11^T, respectively (B).

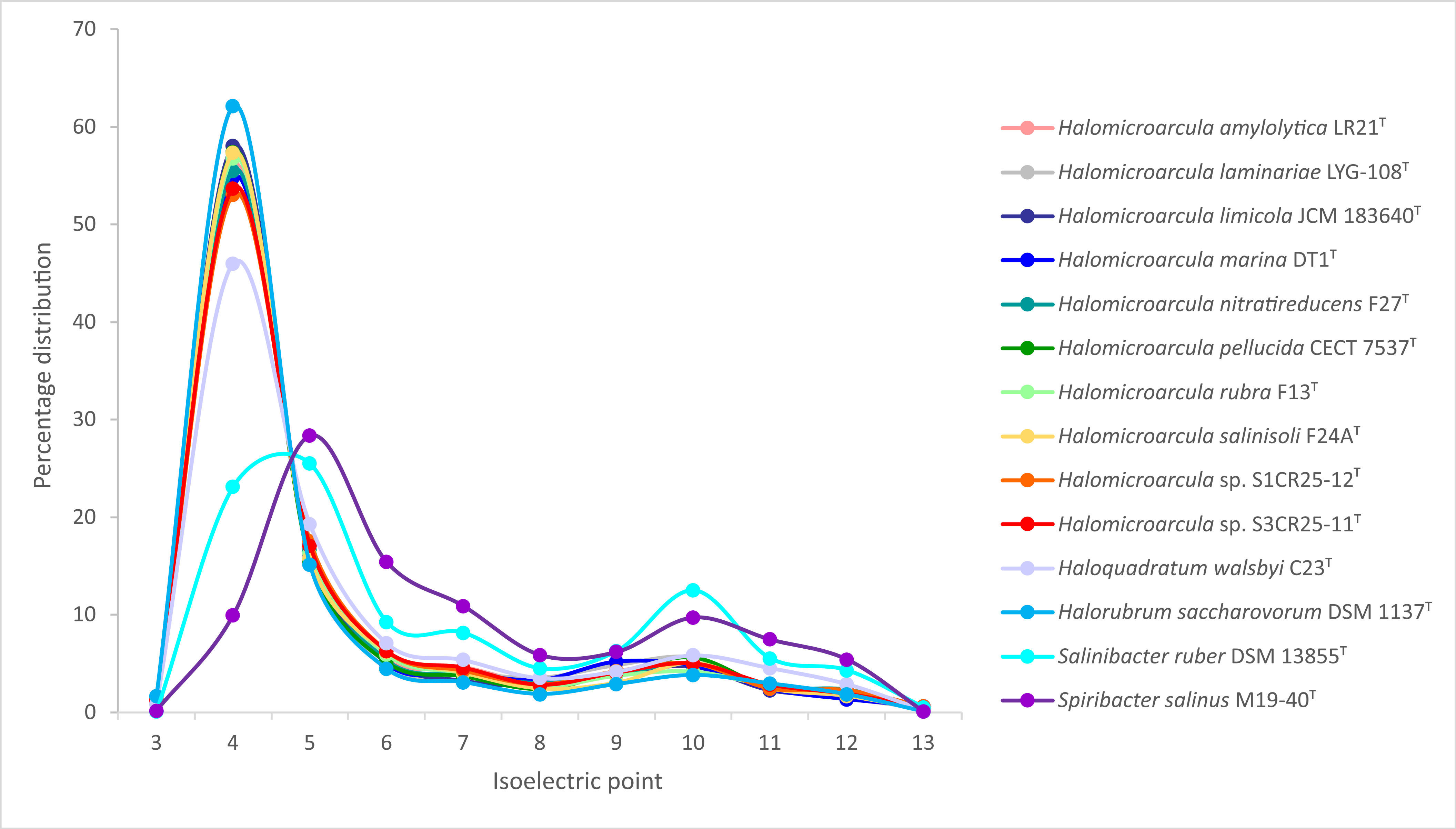

Supplementary Figure S3. Isoelectric point comparison of predicted proteins from the type strains of species of the genus *Halomicroarcula*, including the new isolates S1CR25-12^{T} and S3CR25-11^{T}, and other reference strains.

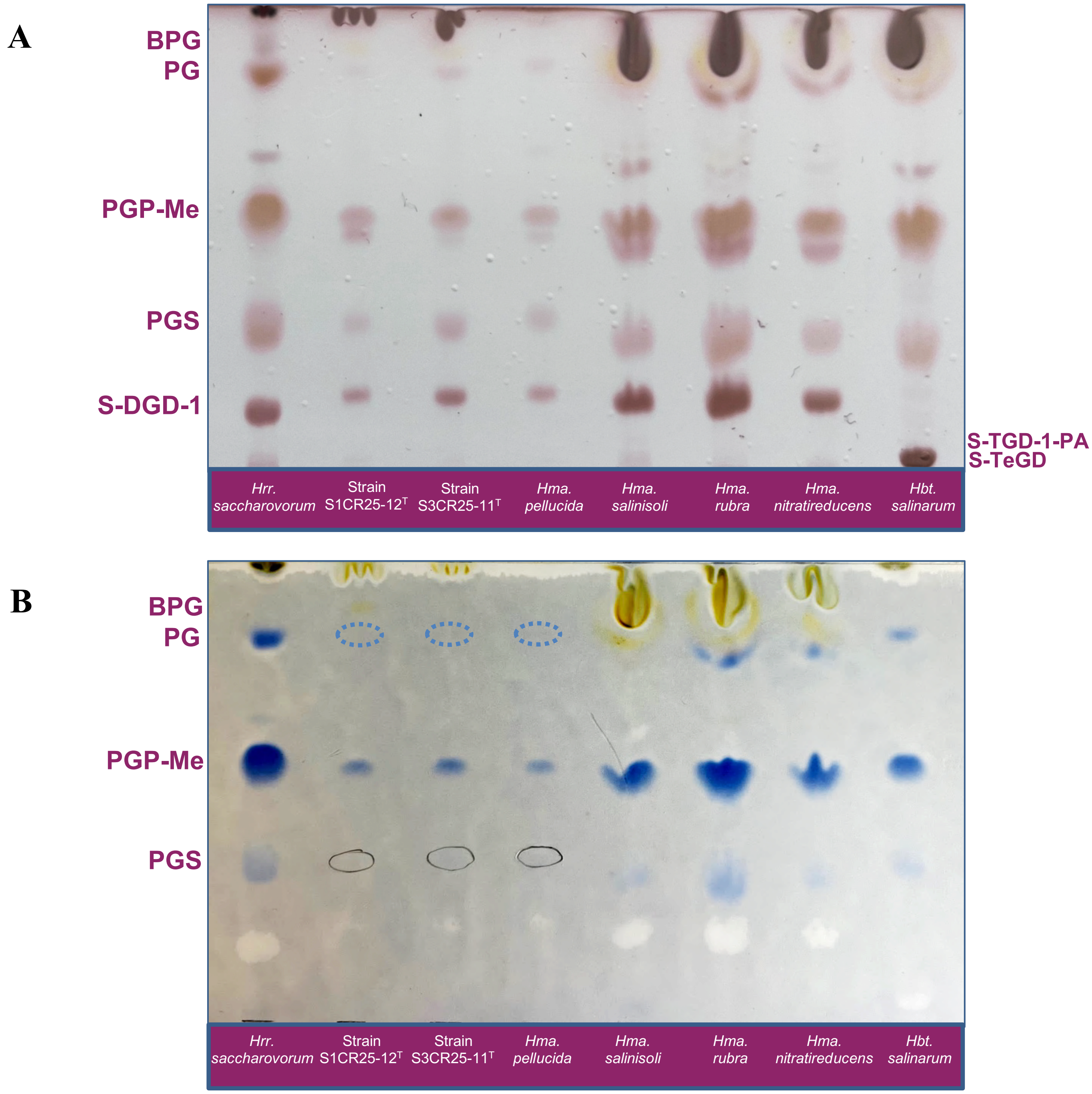

Supplementary Figure S4. High-performance thin-layer chromatography (HPTLC) showing the comparison of the polar lipids (A) and phospholipids (B) profiles between strains S1CR25-12^T, S3CR25-11^T, and other *Halomicroarcula* species as well as *Halorubrum saccharovorum* and *Halobacterium salinarum*. The plates were revealed with sulfuric acid 5 % (v/v) in water followed by heating at 160 °C (A) and with molybdenum blue spray reagent (B). Abbreviations: BPG, biphosphatidylglycerol; PG, phosphatidylglycerol; PGP-Me, phosphatidylglycerol phosphate methyl ester; PGS, phosphatidylglycerol sulfate; S-DGD-1, sulfated diglycosyl diether; S-TGD-1-PA, glycocardiolipin (sulfated triglycosyl diphytanyl archaeol ester linked to phosphatidic acid); S-TeGD, sulfated tetraglycosyl dieter.

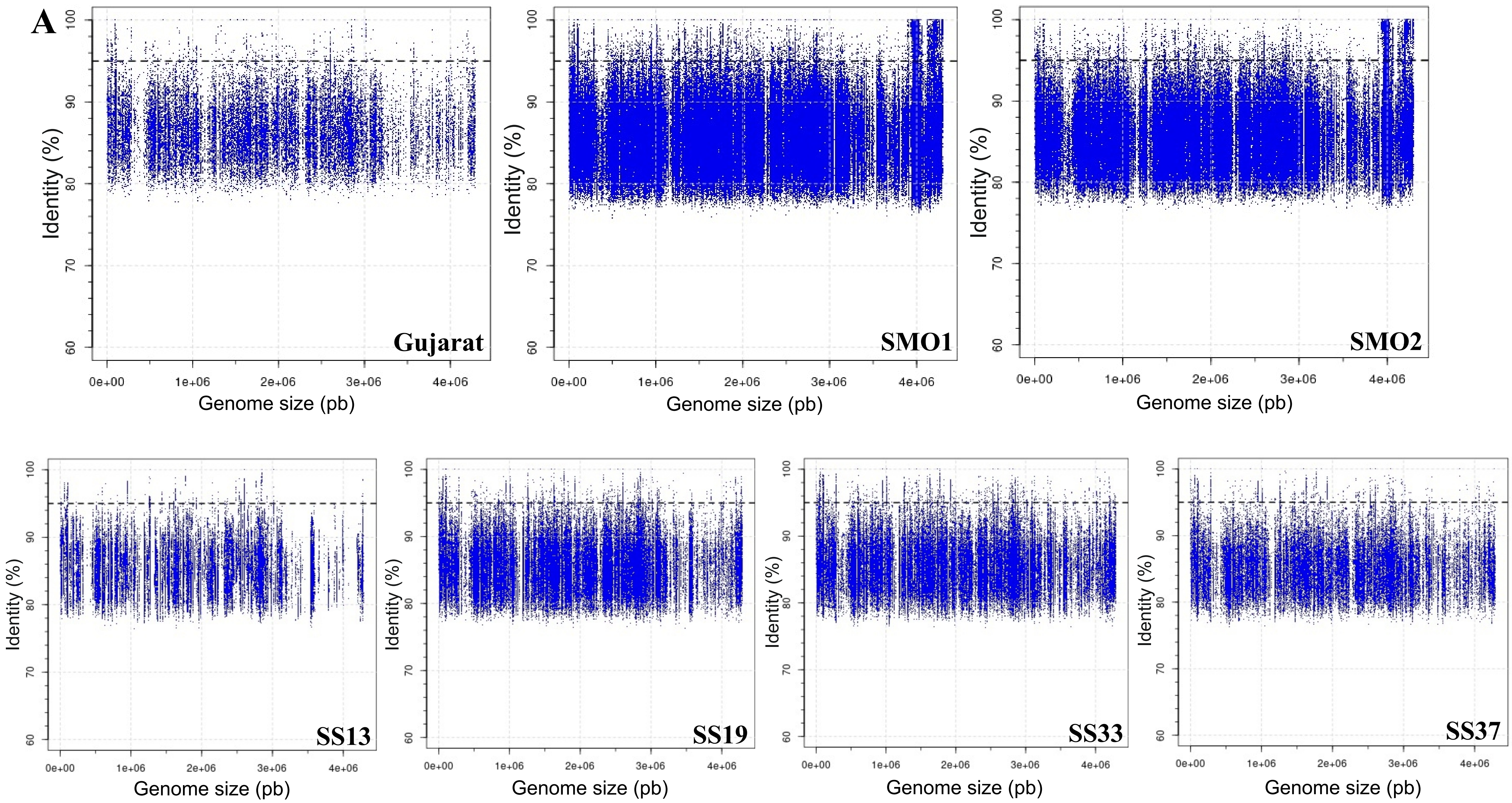
A
Identity (%)
Genome size (pb)
100
90
80
70
60
0e+00
1e+06
2e+06
3e+06
4e+06
Gujarat
SMO1
SMO2
SS13
SS19
SS33
SS37

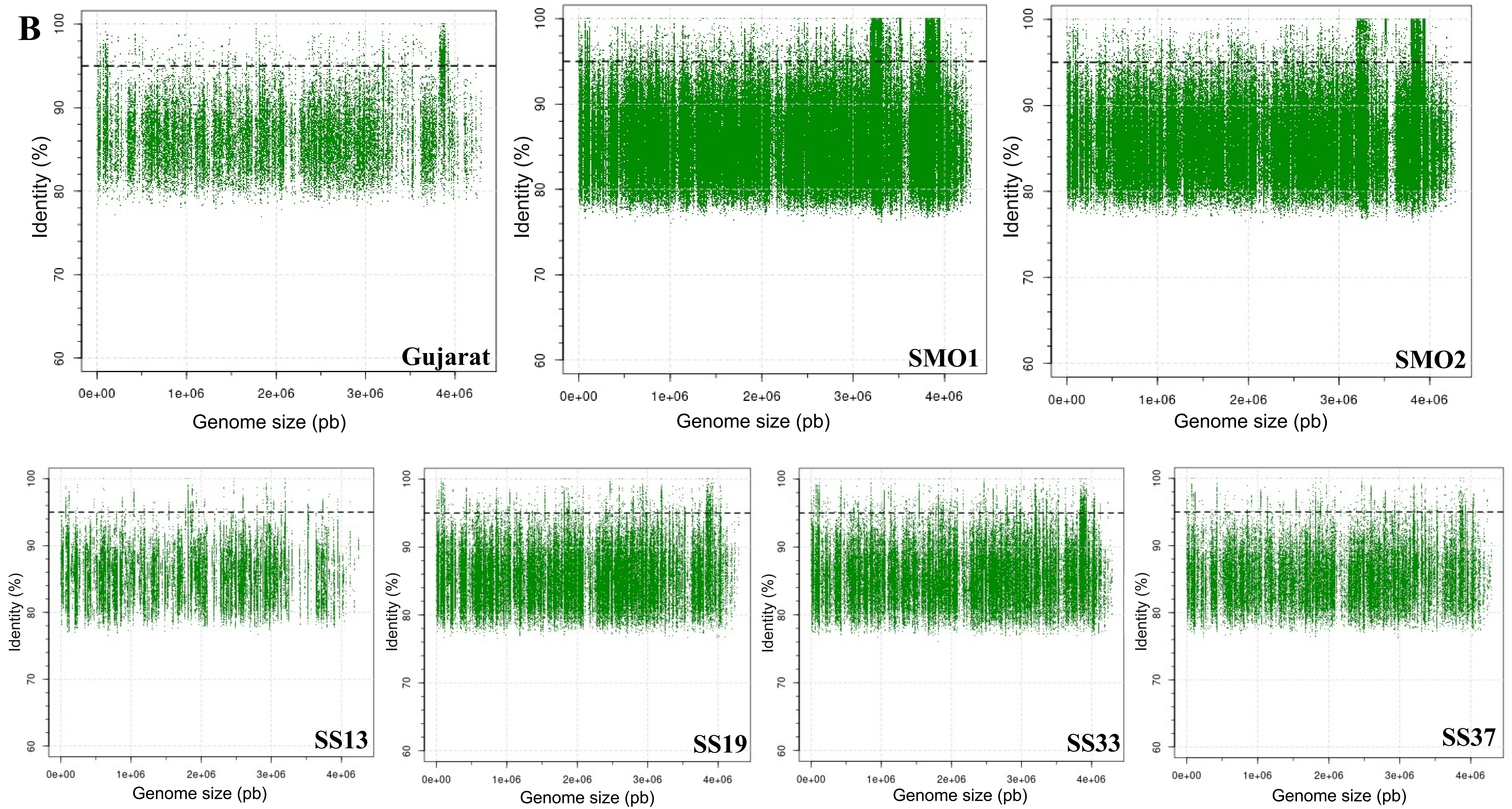

Supplementary Figure S5. Recruitment plots of the two novel strains S1CR25-12^T (A), and S3CR25-11^T (B) against seven different hypersaline metagenomic datasets. Further information about the metagenomes is detailed in Supplementary Table S1. The dashed line shows the threshold for the presence of same species (95% identity).
